# Supplementation with Serum-Derived Extracellular Vesicles Reinforces Antitumor Immunity Induced by Cryo-Thermal Therapy

**DOI:** 10.3390/ijms222011021

**Published:** 2021-10-13

**Authors:** Yinuo Cen, Yue Lou, Junjun Wang, Shicheng Wang, Peng Peng, Aili Zhang, Ping Liu

**Affiliations:** 1 School of Biomedical Engineering, Shanghai Jiao Tong University, Shanghai 200030, China; yinuo.cen@sjtu.edu.cn (Y.C.); LY_017082910045@sjtu.edu.cn (Y.L.); sjtuwangjunjun@sjtu.edu.cn (J.W.); shichengwang@sjtu.edu.cn (S.W.); 112358answer@sjtu.edu.cn (P.P.); zhangaili@sjtu.edu.cn (A.Z.); 2 School of Biomedical Engineering, Med-X Research Institute, Shanghai Jiao Tong University, Shanghai 200030, China

**Keywords:** extracellular vesicle, cryo-thermal therapy, antitumor immunity

## Abstract

Effective cancer therapies should reshape immunosuppression and trigger antitumor immunity. Previously, we developed a novel cryo-thermal therapy through applying local rapid cooling followed by rapid heating of tumor tissue. It could not only ablate local tumors, but also, subsequently, induce systemic long-term antitumor immunity. Hyperthermia can induce the release of extracellular vesicles (EVs) to stimulate antitumor immunity. We examine whether EVs are released after cryo-thermal therapy and whether they could improve the efficacy of cryo-thermal therapy in the 4T1 model. In this study, serum extracellular vesicles (sEVs) are isolated and characterized 3 h after cryo-thermal therapy of subcutaneous tumors. sEV phagocytosis is observed in vitro and in vivo by using laser confocal microscopy and flow cytometry. After cryo-thermal therapy, sEVs are administered to mice via the tail vein, and changes in immune cells are investigated by using flow cytometry. After cryo-thermal therapy, a large number of sEVs are released to the periphery carrying danger signals and tumor antigens, and these sEVs could be phagocytosed by peripheral blood monocytes and differentiated macrophages. After cryo-thermal therapy, supplementation with sEVs released after treatment promotes the differentiation of myeloid-derived suppressor cells (MDSCs), monocytes into macrophages and CD4^+^ T cells into the Th1 subtype, as well as prolonging the long-term survival of the 4T1 subcutaneous tumor-bearing mice. sEVs released after cryo-thermal tumor treatment could clinically serve as an adjuvant in subsequent cryo-thermal therapy to improve the therapeutic effects on malignant tumors.

## 1. Introduction

Major challenges facing ongoing oncologic therapies include metastasis and recurrence, which are mainly caused by malignancy or treatment-induced host immunosuppression [1]. In malignant tumors, immature immune cells are recruited and converted to regulatory subpopulations such as myeloid-derived suppressor cells (MDSCs) and regulatory T cells (Tregs), and these cells promote resistance to therapies and enhance tumor aggressiveness [2,3]. Therefore, the most effective strategies for cancer treatments should alleviate immunosuppression and enhance the host immune response to cancer.

Conventional tumor therapy modalities, such as surgical resection and chemotherapy, often fail to alleviate host immunosuppression and induce effective immune responses [4,5]. In this context, the last decade has witnessed remarkable advances in tumor immunotherapy, which aims to treat malignancies, especially by leveraging the cytotoxic potential of tumor-specific T cells [6,7]. However, immunotherapy cannot relieve tumor-mediated immunosuppression, as most patients experience resistance [8].

In recent years, tumor thermal therapy has become increasingly popular due to its low cost, minimal invasiveness, and strong stimulation of antitumor immunity [9,10]. Effective ablation of local lesions can cause the immunogenic cell death (ICD) of tumor cells [11]. During this process, the released tumor antigens and chaperones recruit innate immune cells and, subsequently, activate T cells, stimulating systemic antitumor immune responses [9,12,13]. Previously, we developed a novel cryo-thermal therapy for tumors through alternative cooling and heating of tumor tissue in animal models [14,15,16]. After cryo-thermal therapy, tumors release tumor antigens and danger signals in situ, which facilitates a durable antitumor adaptive immune response [17,18,19]. In addition to danger signals and tumor antigens, the exposure of tumor cells to hyperthermia can also promote the release of large amounts of extracellular vesicles (EVs), which are phagocytosed by immune cells, leading to the activation of antitumor immunity [20,21,22]. EVs mainly consist of microvesicles and exosomes and carry a large number of nucleic acids, proteins, and other molecules that reflect the state of their parent cells [23,24]. When phagocytosed by antigen-presenting cells, EVs released from heat-shocked tumor cells and ascites can modulate the phenotype and function of innate and adaptive immune cells to promote antitumor immune responses [22,25,26]. However, the release of EVs and their possible immunological properties after cryo-thermal therapy remain to be investigated.

In our previous studies, cryo-thermal therapy achieved satisfactory efficacy in a mouse B16F10 melanoma model [27], but long-term survival remains to be improved in highly tumorigenic and invasive 4T1 mammary carcinoma [18]. The 4T1 murine mammary carcinoma is highly metastatic and immunosuppressive, and the cure for this tumor model requires a strong systemic antitumor immune response [28]. To improve the efficacy of cryo-thermal therapy on malignant 4T1 murine mammary carcinoma, we hypothesized that EVs were released after cryo-thermal therapy into circulation, and that the administration of these released EVs after cryo-thermal therapy could enhance cryo-thermal therapy-induced antitumor immunity and improve survival in 4T1 murine mammary carcinoma.

In this study, serum-derived EVs (sEVs) were isolated and characterized from 4T1 murine mammary carcinoma 3 h after cryo-thermal therapy. After cryo-thermal therapy, additional sEVs released after cryo-thermal therapy were administered intravenously to mice with 4T1 murine mammary carcinoma, and changes in splenic and peripheral immune cells and survival were investigated. This study shows the massive release of sEVs carrying danger signals and tumor-associated antigens. sEVs released after cryo-thermal therapy could be engulfed by monocytes and macrophage-like monocytes (F4/80^+^ Monocytes), and supplementation with sEVs released after cryo-thermal therapy promoted the differentiation of MDSCs and monocytes into macrophages and CD4^+^ T cells into the T helper 1 (Th1) subset in the spleen and peripheral blood. Finally, supplementation with sEVs released after cryo-thermal therapy improved the cryo-thermal-induced long-term survival of tumor-bearing mice. Our study elucidated the antitumor properties of local cryo-thermal therapy-induced sEVs and showed that supplementing serum sEVs released after cryo-thermal therapy could improve the efficacy of cryo-thermal therapy through macrophage and CD4^+^ Th1 cell differentiation.

## 2. Results

### 2.1. Cryo-Thermal Therapy Induces Extracellular Vesicle Release into the Peripheral Circulation

Local tumor hyperthermia can induce the release of EVs bearing tumor antigens and danger signals, which participate in stimulating antitumor immune responses [20]. Previously, we reported an enhanced maturation of macrophages and DCs early after cryo-thermal therapy [18,27]. To investigate whether EVs were related to the maturation of macrophages and DCs, we first investigated the mRNA levels of molecules associated with EV uptake (CD11a, CD54, CD51, CD61, and milk fat globule EGF and factor V/VIII domain containing (MFGE-8)) of these two cell populations [29]. We found an upregulated expression of EV uptake-related genes in both splenic macrophages and DCs (Supplementary Figure S1). Moreover, stronger fold changes in CD11a, CD51, and MFGE-8 were observed in macrophages than in DCs (Supplementary Figure S1). These results suggested that after cryo-thermal therapy, macrophages showed a more robust potential for EV phagocytosis than DCs.

**Figure 1:**
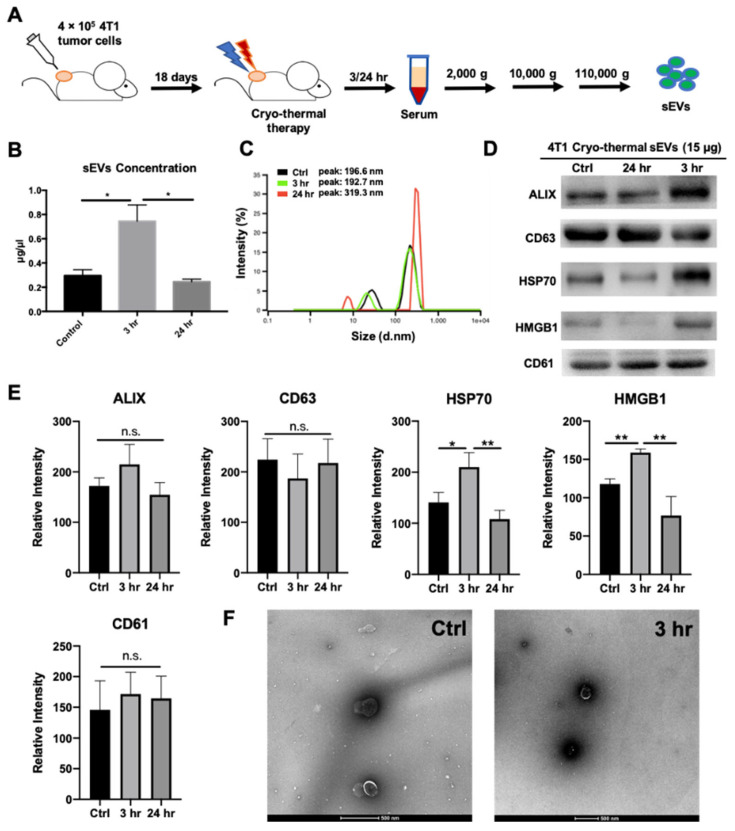
Isolation and characterization of serum extracellular vesicles (sEV) released into periphery after cryo-thermal therapy. (**A**) Schematic of sEV isolation procedures. Approximately 4 × 10^5^ 4T1 cells were injected subcutaneously into the right flank of each mouse; 18 days after tumor inoculation, mice were treated with cryo-thermal therapy. Peripheral blood was collected for serum at 3 and 24 h after treatment and sEVs were isolated by differential centrifugation. (**B**) Protein concentration of sEVs after cryo-thermal therapy was measured with BCA assay. (**C**) Particle size distribution of sEVs isolated from tumor-bearing or cryo-thermal-treated mice at 3 or 24 h after cryo-thermal therapy. Intensity weighted z-average diameters were measured using dynamic light scattering (DLS). (**D**,**E**) Western blotting of sEVs (15 μg) at 3 and 24 h after therapy with individual densitometry analysis. ALIX, CD63: exosomal protein markers; HSP70, HMGB1: danger signals; CD61: platelet marker. (**F**) Morphology of sEVs isolated from tumor-bearing or cryo-thermal-treated mice at 3 h post treatment. Images were obtained using transmission electron microscopy (TEM). Data were shown as mean ± SD, and the Student’s *t*-test was used for statistical analysis. *n* = 4, * *p* < 0.05, ** *p* < 0.01.

Then, we investigated whether EVs were released into the peripheral circulation of mice with 4T1 mammary carcinoma after cryo-thermal therapy. Accordingly, EVs were isolated by differential centrifugation from the serum of cryo-thermal therapy-treated mice at different time points (3 h and 24 h) after treatment (sEVs) (Figure 1A) [30]. Next, the characteristics of sEVs from untreated mice (tumor-bearing control) and treated mice at 3 and 24 h after cryo-thermal therapy were assessed. The BCA assay showed that the highest sEV concentration in serum was observed at 3 h after cryo-thermal therapy compared to that of the tumor-bearing control and treated mice at 24 h (Figure 1B). Particle concentration was measured by the Nanoparticle Tracking Analysis (NTA). Similarly, particle concentration was increased at 3 h after cryo-thermal therapy (Supplementary Figure S2). As shown by dynamic light scattering (DLS), sEVs from 4T1 tumor-bearing and cryo-thermal therapy-treated mice displayed similar size distributions from 100 to 200 nm (Figure 1C). Western blotting showed a similar enrichment in EV-associated proteins ALG-2 interacting protein X (ALIX) and CD63 among the three groups, while a markedly higher expression of danger signaling molecules, such as heat shock protein 70 (HSP70) and high-mobility group box 1 (HMGB1), was found at 3 h after cryo-thermal therapy than in the control and at 24 h after treatment (Figure 1D,E). The level of CD61 (platelet glycoproteins IIIa) in the sEVs from the tumor-bearing control group and cryo-thermal-treated group was also identified (Figure 1D,E). Platelet-derived EVs were part of isolated sEVs. However, unlike the high level of HSP70 and HMGB1 in the sEV at 3 h after cryo-thermal therapy, the level of CD61 carried by sEV had no noticeable change in the tumor-bearing control group, at either 3 h or 24 h after cryo-thermal therapy (Figure 1E). Therefore, CD61 would not play a significant role in cryo-thermal-induced antitumor immunity. To explore whether sEVs could also transport tumor antigens, we repeated the isolation and characterization procedure on a B16F10 melanoma subcutaneous tumor model that is known to express tumor antigen tyrosinase-related protein 2 (TRP2) [19]. Indeed, TRP2 was found in sEVs at different time points (3 h, 12 h, and 24 h) after cryo-thermal therapy by using Western blotting, and the concentration peaked at 3 h after therapy. The expression of other proteins showed similar trends as the 4T1 mammary carcinoma model (Supplementary Figure S3). Finally, TEM images confirmed the similar morphology of sEVs before and at 3 h after treatment (Figure 1F). Taken together, these results indicated that cryo-thermal therapy could induce the release of sEVs carrying danger signals and tumor antigens into peripheral circulation. The highest protein and particle concentrations of sEVs carrying tumor antigens and danger signals occurred at 3 h after cryo-thermal therapy.

### 2.2. Uptake of Cryo-Thermal sEVs into Circulating Monocytes and Macrophage-Like Monocytes

**Figure 2:**
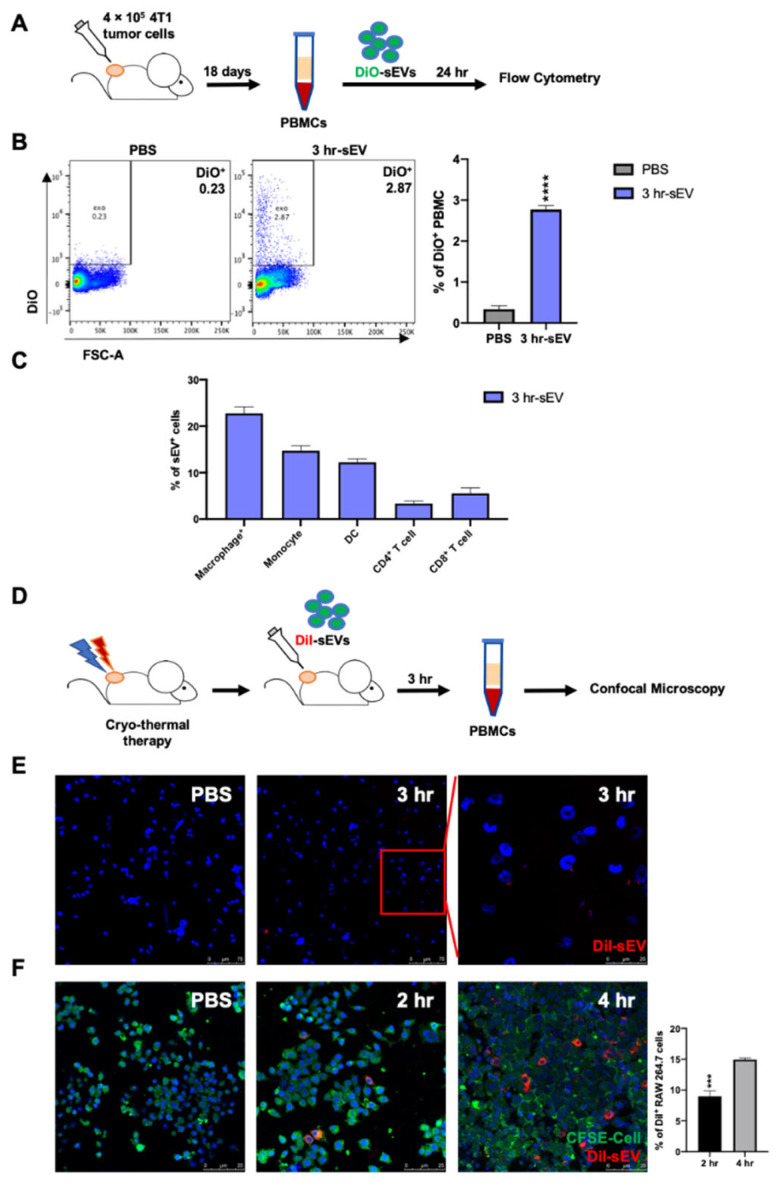
Phagocytosis of sEVs. (**A**) Schematic of experimental design. sEVs were labeled by DiO and PBS was dyed, diluted, and centrifuged as negative control. PBMCs isolated from tumor-bearing mice were cultured with DiO-labeled sEVs at 20 μg/mL or PBS in vitro for 24 h. The proportion of phagocytosis of sEVs in PBMCs was detected by using flow cytometry. (**B**) The proportion of phagocytosis of sEVs by PBMCs. (**C**) The proportion of macrophages (F4/80^+^CD11b^+^), monocytes (Ly6G^−^Ly6C^hi^CD11b^+^), DCs (CD11c^hi^), CD4^+^ T cells (CD3^+^CD4^+^), and CD8^+^ T cells (CD3^+^CD8^+^) of 3 hr-sEV positive PBMCs. (**D**) Schematic of experimental design. DiI-labeled sEVs were injected into mice immediately after cryo-thermal therapy via the tail vein; 3 h later, PBMCs were isolated and observed by using confocal fluorescence microscope. (**E**) Qualitatively displayed that some cells with large kidney-shaped nuclei in PBMCs were co-located with sEVs. PBMCs isolated from cryo-thermal therapy-treated mice with or without sEVs supply (50 μg) i.v. injection were fixed on adhesion slides and analyzed by using confocal microscopy. In the overlay, sEVs appear as red dots and were co-located with cells of large, kidney-shaped nuclei. (**F**) Uptake of sEVs by RAW 264.7 cells (20 μg/mL). CFSE-labeled RAW264.7 cells were incubated with DiI-labeled sEVs for 2 or 4 h. Cells were fixed on adhesion slides and analyzed by using confocal microscopy. In the overlay, serum sEVs appear as red dots and the uptake was quantitated using ImageJ. Data were shown as mean ± SD, and the Student’s *t*-test was used for statistical analysis. *n* = 4, *** *p* < 0.001, **** *p* < 0.0001.

We hypothesized that peripheral blood mononuclear cells (PBMCs) would phagocytize the released sEVs after cryo-thermal therapy. To investigate whether sEVs could be directly taken up by PBMCs after cryo-thermal therapy, PBMCs were isolated from 4T1 tumor-bearing mice and cultured with DiO-labeled sEVs for 24 h. Then, the PBMCs were analyzed by flow cytometry (Figure 2A). The proportion of DiO^+^ PBMCs was significantly higher than that in the PBS group, and there was a massive engulfment of sEVs by PBMCs (Figure 2B). Among the identified sEV-positive phagocytic cells, more than 20% of all phagocytic cells were derived macrophages, which was much higher than the proportions of monocytes and DCs (Figure 2C). To directly verify the phagocytosis of mononuclear phagocytes in vivo, DiI-labeled sEVs were injected into 4T1 tumor-bearing mice immediately after cryo-thermal therapy via the tail vein to mimic local sEV release into peripheral circulation (Figure 2D). Three hours later, PBMCs were isolated and observed by confocal microscopy. Consistently, Figure 2E qualitatively displayed that some cells with large kidney-shaped nuclei in PBMCs were co-located with sEVs (Figure 2E). Given that after cryo-thermal therapy sEVs were mainly engulfed by monocytes and macrophage-like monocytes (F4/80^+^ Monocytes), the RAW264.7 macrophage cell line was used to investigate the uptake of sEVs. After coincubation with sEVs from 2 to 4 h, CFSE-labeled RAW264.7 cells were colocalized with DiI-labeled sEVs (Figure 2F), and the uptake of sEVs by RAW264.7 cells at 4 h was much stronger than that at 2 h of coculture. These results showed that sEVs could be phagocytosed by PBMCs, and they were mainly engulfed by monocytes and macrophage-like monocytes in the peripheral blood of 4T1 tumor-bearing mice.

### 2.3. sEVs Released after Cryo-Thermal Therapy Potentiated the Activation of Innate Immunity Induced by Cryo-Thermal Therapy

**Figure 3:**
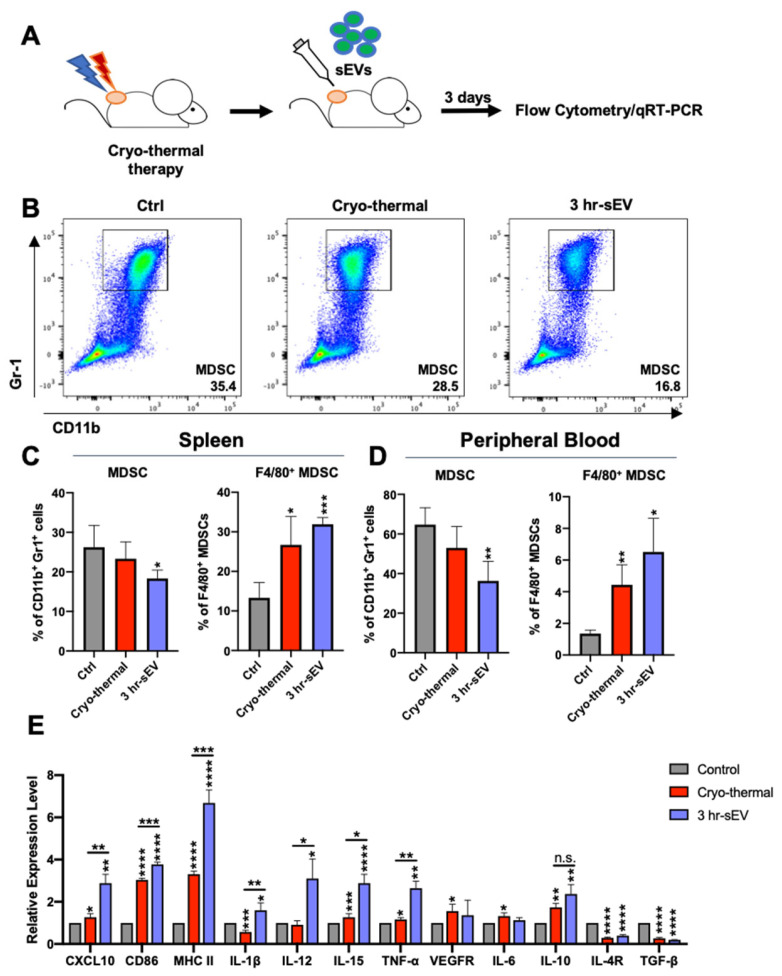
Supplement of sEVs after cryo-thermal therapy promoted the differentiation of MDSCs and monocytes to macrophages in PBMCs. (**A**) Schematic of experimental design; 50 μg of sEVs was injected into mice immediately after cryo-thermal therapy via the tail vein; 3 days later, splenocytes and PBMCs were isolated and characterized by using flow cytometry. (**B**) Flow cytometry gating strategy for determination of CD11b^+^ Gr1^+^ MDSCs. (**C**,**D**) Proportions of MDSCs and F4/80^+^ MDSCs in spleen (**C**) and peripheral blood (**D**) were detected by flow cytometry, and the Student’s *t*-test was used for statistical analysis (*n* = 4 per group). (**E**) Immune-related molecules of MDSCs were examined by qRT-PCR on day 3 after cryo-thermal therapy and sEV injection (*n* = 4 per group). Data were shown as mean ± SD. * *p* < 0.05, ** *p* < 0.01, *** *p* < 0.001, **** *p* < 0.0001 by one-way ANOVA.

Extracellular vesicle release can induce systemic antitumor immunity in response to thermal physical therapies [20]. After cryo-thermal therapy, sEVs carrying danger signals and tumor antigens were released into the peripheral circulation and were taken up by monocyte lineage cells, which might alter the immunostimulatory profile of innate and adaptive immune cells; thus, triggering antitumor immunity. In this context, we examined whether the released sEVs could improve the efficacy of cryo-thermal therapy in highly tumorigenic and invasive 4T1 mammary carcinoma. The released sEVs in the peripheral blood of 4T1 tumor-bearing mice were collected at 3 h after cryo-thermal therapy. In total, 50 μg sEVs was then injected into 4T1 tumor-bearing mice through the tail vein immediately after cryo-thermal therapy, and the status of immune cells in the spleen and peripheral blood on day 3 after treatment was determined by using flow cytometry (Figure 3A).

Innate immunity plays a critical and acute role in various antitumor therapies [31]. Previous studies reported early macrophage M1 polarization and DC maturation after cryo-thermal therapy [18,27]. Likewise, the proportions of macrophages in the spleen were obviously increased on day 3 after cryo-thermal therapy (Supplementary Figure S4A,B). In the peripheral blood, the proportion of macrophage-like monocytes was upregulated on day 3 after cryo-thermal therapy alone or cryo-thermal therapy combined with sEV injection compared to that of the tumor-bearing control group (Supplementary Figure S4C). However, neither cryo-thermal therapy alone nor cryo-thermal therapy combined with sEV injection altered the levels of the costimulatory molecule CD86 and antigen presentation-related protein MHC II (Supplementary Figure S4B,C). On day 3 after cryo-thermal therapy, there were light increases in the proportion of splenic DCs and the level of CD86 in DCs. However, cryo-thermal therapy combined with sEV injection did not affect the proportion or maturation signature of DCs (Supplementary Figure S4D–F).

MDSCs can drive tumor immune escape by suppressing the adaptive immune response of T cells [32]. The infiltration of MDSCs is causally related to the malignancy of the 4T1 mammary carcinoma model [33]. On day 3 after cryo-thermal therapy, the proportion of MDSCs in the spleen did not obviously change, but the expression of the macrophage marker F4/80 in MDSCs was slightly higher than that in the tumor-bearing control group (Figure 3B,C). On day 3 after cryo-thermal therapy combined with sEV injection, the proportion of MDSCs was decreased compared to that of the tumor-bearing control group. Notably, cryo-thermal therapy combined with sEV injection elicited a stronger increase in F4/80 expression in MDSCs than that in the cryo-thermal group (Figure 3B,C). A similar trend was observed in PBMCs (Figure 3D).

We further isolated spleen MDSCs from mice on day 3 after cryo-thermal therapy alone or cryo-thermal therapy with sEV injection and examined the expression of immune-related molecules in MDSCs by qRT-PCR (Figure 3E). The levels of molecules representing the function of M1 macrophages, such as CD86, MHC II, CXCL10, and IL-12, were markedly increased in the cryo-thermal therapy with sEV injection group compared to that from the tumor-bearing and cryo-thermal therapy-treated mice (Figure 3E). Consistently, the expression of inflammatory cytokines, such as IL-1β, IL-12, IL-15, and TNF-α in MDSCs, was significantly increased in cryo-thermal therapy with the sEV injection group (Figure 3E). On the other hand, the expression of immunosuppressive molecules in MDSCs, including IL-4R and TGF-β, was markedly decreased in the cryo-thermal therapy alone and combination of cryo-thermal and sEV injection group (Figure 3E). The level of VEGFR and IL-6 in MDSCs only slightly increased in the cryo-thermal group (Figure 3E). The expression level of VEGFR and IL-6 in the sEV supplemented group had no noticeable change compared to the tumor-bearing control group. Though the level of IL-10 was upregulated in mice treated with cryo-thermal therapy only and a combination of cryo-thermal treated and sEV supplemented mice, there was no significant difference between them (Figure 3E). Therefore, cryo-thermal therapy with sEV injection could abolish the immunosuppressive properties of MDSCs and enhance the expression of molecules representing functions and inflammatory cytokines of macrophages, which indicated that cryo-thermal therapy with sEV injection could drive MDSCs maturation into macrophages.

**Figure 4:**
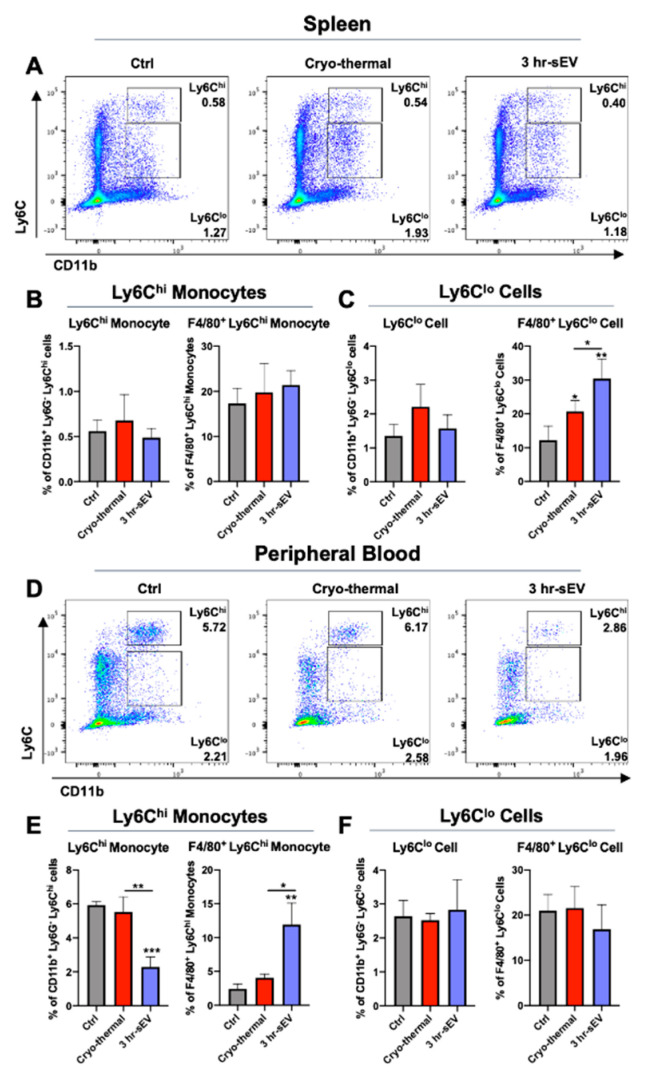
Supplement of sEVs after cryo-thermal therapy promoted the differentiation of monocytes to macrophages. (**A**,**D**) Flow cytometry gating strategy for determination of CD11b^+^ Ly6C^hi^ monocytes and CD11b^+^ Ly6C^lo^ monocytes in Ly6G^−^ cells in spleen (**A**) and peripheral blood (**D**). (**B**,**C**) Proportions of Ly6C^hi^ monocytes (**B**), Ly6C^lo^ monocytes (**C**) and their expression of F4/80 in spleen. (**E**,**F**) Proportions of Ly6C^hi^ monocytes (**E**), Ly6C^lo^ monocytes (**F**) and their expression of F4/80 in peripheral blood were detected by flow cytometry. *n* = 4 per group. Data were shown as mean ± SD, and the Student’s *t*-test was used for statistical analysis. * *p* < 0.05, ** *p* < 0.01, *** *p* < 0.001.

Monocytes constitute the vital pool of derived macrophages [34]. Ly6C^hi^ monocytes and Ly6C^lo^ cells were separately analyzed (Figure 4). Both Ly6C^hi^ monocytes and Ly6C^lo^ cells in the spleen expressed F4/80. The proportion of Ly6C^hi^ monocytes and Ly6C^lo^ cells in the spleen remained steady on day 3 after cryo-thermal therapy alone or cryo-thermal therapy combined with sEV injection. There was no significant difference in the expression of the macrophage marker F4/80 in Ly6C^hi^ monocytes between the tumor-bearing and treated mouse groups (Figure 4A–C). However, in Ly6C^lo^ cells, cryo-thermal therapy alone significantly upregulated the expression of the macrophage marker F4/80. Moreover, cryo-thermal therapy combined with sEV injection further increased the expression of the macrophage marker F4/80 in Ly6C^lo^ cells, which was much higher than that of the cryo-thermal therapy alone group (Figure 4C).

In the peripheral blood, the percentage of Ly6C^hi^ monocytes was down-regulated after cryo-thermal therapy alone or with sEV supplementation (Figure 4D–F). Compared to cryo-thermal therapy alone, the supplementation of sEVs further reduced the proportion of Ly6C^hi^ monocytes in the blood (Figure 4D,E). Of notice, the combination of cryo-thermal therapy and sEV supplementation induced a significantly high expression of F4/80 in Ly6C^hi^ monocytes (Figure 4D,E). The proportion of Ly6C^lo^ cells and their expression of F4/80 in peripheral blood remained unchanged on day 3, after cryo-thermal therapy alone or cryo-thermal therapy combined with sEV injection (Figure 4F).

These results suggested that supplementation of sEVs could promote the differentiation of monocytes into macrophage-like monocytes in peripheral blood and Ly6C^lo^ cells into macrophages in the spleen.

### 2.4. sEVs Released after Cryo-Thermal Therapy Promoted the Differentiation of CD4^+^ T Cells into the Th1 Subset

In addition to innate immune cells, EVs can also trigger antitumor immunity by activating T cells [22]. To explore the effect of the released sEVs after cryo-thermal therapy on lasting antitumor immunity induced by cryo-thermal therapy, we analyzed the percentages of CD4^+^ T cells in the spleen and peripheral blood on day 3 after treatments. Additionally, CD4^+^ T cells exhibit high plasticity and can differentiate into multiple subtypes in response to developmental and environmental cues [35]. Therefore, the changes in CD4^+^ T cell subsets in the spleen and peripheral blood were also explored by using flow cytometry.

**Figure 5:**
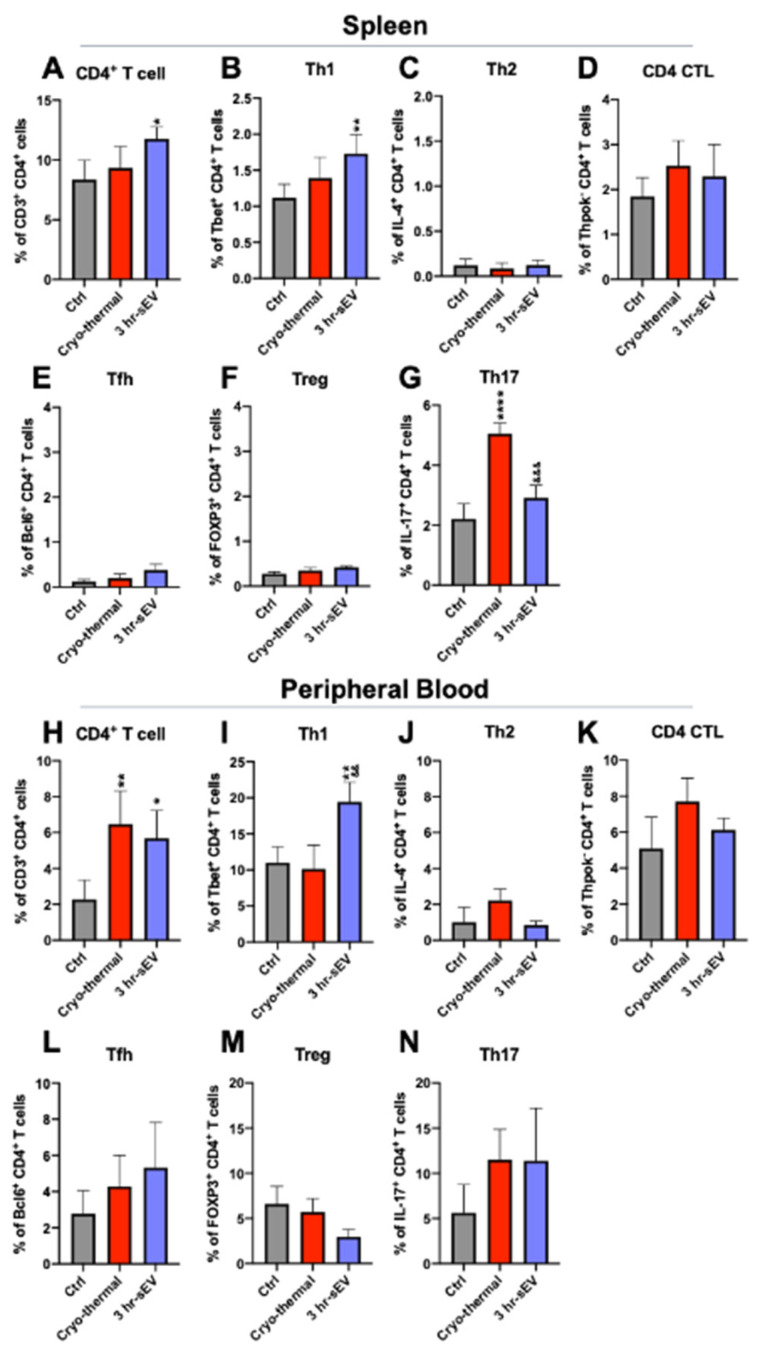
The released sEVs after cryo-thermal therapy promoted the differentiation of CD4^+^ T cells into Th1 subtype. Flow cytometry was used to determine the ratio of CD4^+^ T cells in the spleen (**A**–**G**) and peripheral blood (**H**–**N**) on day 3 after cryo-thermal therapy. (**A**–**G**) The proportions of CD4^+^ T cells (**A**), Th1 (T-bet^+^) (**B**), Th2 (IL-4^+^) (**C**), CD4^+^ CTL (Thpok-) (**D**), Tfh (Bcl6^+^) (**E**), Tregs (FOXP3^+^) (**F**), and Th17 (IL-17^+^) (**G**) in spleen were determined by using flow cytometry (*n* = 4 per group). (**H**–**N**) The proportions of CD4^+^ T cells (**H**), Th1 (T-bet^+^) (**I**), Th2 (IL-4^+^) (**J**), CD4^+^ CTL (Thpok-) (**K**), Tfh (Bcl6^+^) (**L**), Tregs (FOXP3^+^) (**M**), and Th17 (IL-17^+^) (**N**) in peripheral blood were determined by using flow cytometry (*n* = 4 per group). Data were shown as mean ± SD, and the Student’s t-test was used for statistical analysis. *n* = 4, * *p* < 0.05, ** *p* < 0.01, **** *p* < 0.0001 compared with the control group; && *p* < 0.01; &&& *p* < 0.001 compared with the cryo-thermal treatment group.

A flow cytometry analysis showed that the proportion of CD4^+^ T cells in the spleen was increased after cryo-thermal therapy combined with sEV injection compared to that in the tumor-bearing group (Figure 5A). Supplementation with sEVs after cryo-thermal therapy could significantly upregulate the ratio of the Th1 subset, as indicated by a significant increase in the expression of the transcription factor T-bet (Figure 5B). In addition, the percentage of Th17 cells in the spleen increased markedly after cryo-thermal therapy, while supplementation with sEVs after cryo-thermal therapy inhibited the cryo-thermal therapy-induced level of Th17 cells (Figure 5G). In the peripheral blood, both cryo-thermal therapy alone and cryo-thermal therapy combined with sEV injection upregulated the proportion of CD4^+^ T cells (Figure 5H); however, only supplementation with sEVs after cryo-thermal therapy markedly increased the proportion of the Th1 (T-bet) subset compared with that in the control group and the cryo-thermal group (Figure 4I). The proportions of other CD4^+^ T cell subsets, including CD4 CTLs, Th2 cells (IL-4), Tfhs, and Tregs, in the spleen and peripheral blood showed no obvious changes among the three groups (Figure 5B–N). Collectively, supplementation with sEVs after cryo-thermal therapy promoted the differentiation of CD4^+^ T cells into Th1 cells on day 3 after treatment.

### 2.5. sEVs Released after Cryo-Thermal Therapy Enhanced the Cytotoxicity of CD8^+^ T Cells Induced by Cryo-Thermal Therapy

We also examined changes in CD8^+^ T cells after cryo-thermal therapy combined with sEV injection. The function of CD8^+^ T cells is characterized by the secretion of cytotoxic factors, granzyme B, and perforin [36]. Therefore, the proportion of CD8^+^ T cells and their cytotoxic signature were analyzed by flow cytometry on day 3 after the treatments.

**Figure 6:**
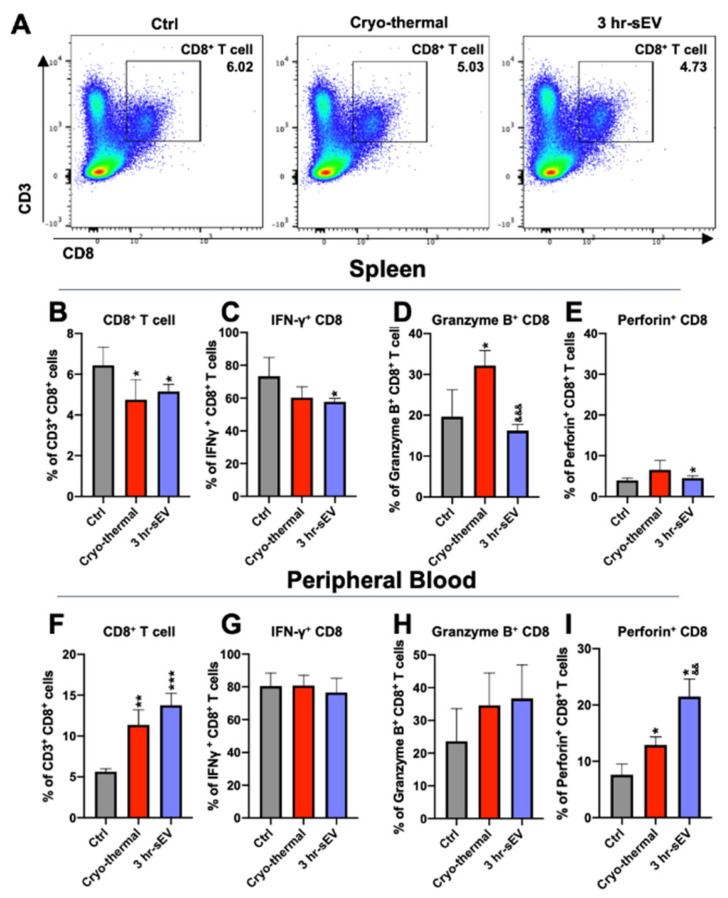
The released sEVs after cryo-thermal therapy promoted the production of cytotoxic CD8^+^ T cells. (**A**) Flow cytometry gating strategy for the determination of CD3^+^ CD8^+^ T cells. (**B**–**E**) The proportions of CD8^+^ T cells (**B**), IFN-γ^+^ CD8^+^ T cells (**C**), Granzyme B^+^ CD8^+^ T cells (**D**), and Perforin^+^ CD8^+^ T cells (**E**) were determined by flow cytometry. (**F**–**I**) The proportions of CD8^+^ T cells (**F**), IFN-γ^+^ CD8^+^ T cells (**G**), Granzyme B^+^ CD8^+^ T cells (**H**), and Perforin^+^ CD8^+^ T cells (**I**) were determined by using flow cytometry (*n* = 4 per group). Data were shown as mean ± SD, and the Student’s *t*-test was used for statistical analysis. *n* = 4, * *p* < 0.05, ** *p* < 0.01. *** *p* < 0.001 compared with the control group; && *p* < 0.01; &&& *p* < 0.001 compared with the cryo-thermal treatment group.

The results showed that the proportion of CD8^+^ T cells was moderately reduced in the spleen after cryo-thermal therapy or cryo-thermal therapy combined with sEV injection compared with that of tumor-bearing mice (Figure 6A,B). After cryo-thermal therapy, the level of Granzyme B in the spleen was increased compared to that in the tumor-bearing control group (Figure 6D). Supplementation with sEVs after cryo-thermal therapy slightly impaired IFN-γ production and enhanced perforin expression in CD8^+^ T cells (Figure 6C–E). In the peripheral blood, the proportion of CD8^+^ T cells was markedly increased after cryo-thermal therapy compared to that of the tumor-bearing control group and was further upregulated when sEVs were administered after cryo-thermal therapy (Figure 6F). The levels of IFN-γ and Granzyme B remained unaltered after therapy with or without sEV supplementation (Figure 6G,H). The level of perforin was slightly increased after cryo-thermal therapy compared to that in the cryo-thermal group and was further upregulated in CD8^+^ T cells after cryo-thermal therapy combined with sEV injection (Figure 6G–I). Therefore, the sEVs released after cryo-thermal therapy could enhance the cytotoxic properties of CD8^+^ T cells in peripheral blood.

### 2.6. sEVs Released after Cryo-Thermal Therapy Promoted Macrophage Maturation and Th1 Cell Differentiation In Vitro

**Figure 7:**
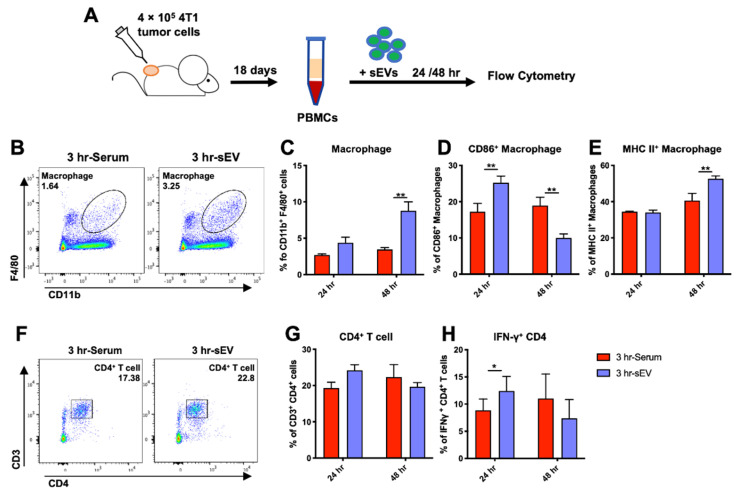
sEVs released after cryo-thermal therapy promoted the maturation of macrophage-like monocytes and CD4^+^ T differentiation toward Th1 in vitro. (**A**) Schematic of experimental design. PBMCs from tumor-bearing mice were incubated with sEVs derived from cryo-thermal treated mice at 20 μg/mL or PBS in vitro for 24 or 48 h. (**B**) Flow cytometry gating strategy for determination of CD11b^+^ F4/80^+^ macrophages in PBMCs. (**C**–**E**) The proportions of macrophages (**C**), CD86^+^ macrophages (**D**), and MHC II^+^ macrophages (**E**) were determined by using flow cytometry. (**F**) Flow cytometry gating strategy for the determination of CD3^+^ CD4^+^ T cells in PBMCs. (**G**,**H**) The proportions of CD4^+^ T cells (**G**) and IFN-γ^+^ CD4^+^ T cells (**H**) were determined by using flow cytometry. Data were shown as mean ± SD, and the Student’s *t*-test was used for statistical analysis. *n* = 4, * *p* < 0.05, ** *p* < 0.01 compared with the control group.

To further verify the underlying effect of sEVs on macrophages and CD4^+^ T cell differentiation after cryo-thermal therapy in vivo, PBMCs from 4T1 tumor-bearing mice were cocultured with sEVs released 3 h after cryo-thermal therapy in vitro for 24 to 48 h (Figure 7A). Serum collected from cryo-thermal therapy-treated mice 3 h after therapy was added to the medium to simulate the corresponding inflammatory environment. Likewise, the proportion of F4/80^+^ monocytes increased sharply at 48 h after coincubation with sEVs (Figure 7B,C). The expression of CD86 was upregulated at 24 h but downregulated at 48 h after coculture compared to that in the group without sEVs (Figure 7D). The level of MHC II markedly increased at 48 h (Figure 7E). The proportion of CD4^+^ T cells did not significantly change after coincubation with sEVs (Figure 7F,G), but the expression of the Th1-related cytokine IFN-γ was markedly increased at 24 h after coculture compared to that in the serum group (Figure 7H). The in vitro studies also showed that sEVs could stimulate macrophage maturation and CD4^+^ T cell differentiation into the Th1 subset.

### 2.7. sEVs Released after Cryo-Thermal Therapy Prolonged the Survival of Mice Treated with Cryo-Thermal Therapy

In vivo and in vitro studies showed that after cryo-thermal therapy, supplementing the treated mice with sEVs could further promote the differentiation of MDSCs and monocytes into macrophages and CD4^+^ T cells into Th1 subtypes.

**Figure 8:**
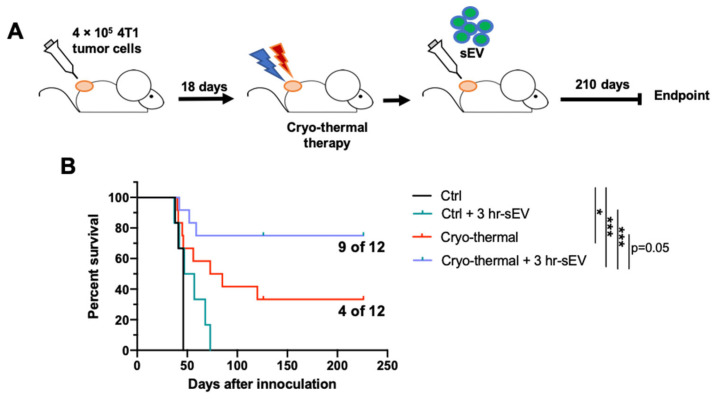
Supplementation with sEVs released after cryo-thermal therapy prolonged the survival of cryo-thermal therapy-treated mice. (**A**) Schematic of experimental design; 4 × 10^5^ 4T1 cells were injected subcutaneously into the right flank. Mice were treated with cryo-thermal therapy 18 days after tumor inoculation; 50 μg sEVs or PBS of the same volume were given to each mouse through the tail vein immediately after cryo-thermal therapy. (**B**) Kaplan–Meier survival plot of the survival observation. Curves were compared using log-rank tests. *n* = 6 for control and control + 3 h-sEV group. *n* = 12 for cryo-thermal and cryo-thermal + 3 h-sEV group. * *p* < 0.05, *** *p* < 0.001.

Finally, to explore whether sEVs could improve the efficacy of cryo-thermal therapy on malignant tumors, we established a highly metastatic 4T1 murine breast subcutaneous tumor model. After therapy, sEVs were administered immediately through the tail vein, and long-term survival (210 days) was observed (Figure 8A,B). As shown in Figure 8, all mice from tumor-bearing control groups were sacrificed because of the primary tumor size and lung metastasis. Only inoculation of sEVs released after cryo-thermal therapy was insufficient to induce effective antitumor immunity, though it slightly elongated the survival of tumor-bearing mice. After cryo-thermal therapy alone, the long-term (210 days) survival rate remained unsatisfactory (4/12: 33%), while the combination of sEVs and cryo-thermal therapy improved the survival significantly (9/12: 75%). Of notice, the difference between the survival curves of cryo-thermal therapy alone and cryo-thermal therapy combined with sEV supplementation reached statistic difference (*p* = 0.05). It was evident that cryo-thermal therapy combined with sEV injection, compared to cryo-thermal therapy alone, achieved a much higher long-term survival rate (9/12: 75%).

Conclusively, supplementation with sEVs released after therapy could further improve the efficacy of cryo-thermal therapy on 4T1 murine breast subcutaneous tumors and prolong the survival times of tumor-bearing mice.

## 3. Discussion

Previously, we reported a profound immunostimulatory and antitumor effect of cryo-thermal therapy on innate and adaptive immunity [18,19,27]. A high survival rate was achieved in the mouse melanoma B16F10 model [20]. However, the efficacy of cryo-thermal therapy in the 4T1 murine mammary carcinoma model still remains to be improved. The 4T1 murine mammary carcinoma is a highly metastatic and immunosuppressive malignancy and the progressive spread of 4T1 metastases to the draining lymph nodes and other organs is very similar to that of human mammary cancer [28]. Therefore, to improve the efficacy of cryo-thermal therapy in the 4T1 murine mammary carcinoma model, it is desirable to enhance immunogenicity leading to trigger a stronger systemic antitumor immunity. Studies have shown that EVs bearing tumor antigens and danger signals released from heat-stressed tumor cells can effectively stimulate antitumor immunity [20,21,22,25,26]. Therefore, to enhance tumor immunogenicity in mice with 4T1 murine mammary carcinoma after cryo-thermal therapy, we sought to investigate whether sEVs were released after cryo-thermal therapy and whether supplementation with sEVs released after cryo-thermal therapy could improve the antitumor immune response and achieve a good therapeutic effect in a 4T1 murine mammary carcinoma model. In this study, mice with highly tumorigenic and invasive 4T1 murine mammary carcinoma were treated with cryo-thermal therapy. After 3 h, a high concentration of sEVs carrying tumor antigens and danger signals was released into the peripheral circulation. The released sEVs were mainly phagocytosed by peripheral blood monocytes and macrophage-like monocytes. After cryo-thermal therapy, supplementation with additional sEVs released after therapy could decrease the level of MDSCs and facilitate the differentiation of MDSCs and monocytes into macrophages and CD4^+^ T cells into the Th1 subtype. Moreover, supplementation with sEVs released after cryo-thermal therapy could prolong the long-term survival of 4T1 tumor-bearing mice after treatment.

The mononuclear phagocyte system (MPS) has a strong phagocytic effect on sEVs [37]. Monocytes play roles in antigen-related phagocytosis and innate immunity [34]. These cells can differentiate into tissue-resident macrophages under proinflammatory conditions [38,39]. In this study, splenic macrophages highly expressed molecules associated with EV phagocytosis, and sEVs released after therapy were mainly engulfed by macrophage-like monocytes and monocytes in the periphery on day 3 after cryo-thermal therapy. The percentage of EVs^+^ cells in CD4^+^ and CD8^+^ T cells was less than 5% in total EVs^+^ cells. sEVs could be internalized by T cells [40,41], and it was also possible that “EVs” were only attached to T cell membranes. A dominant uptake of sEVs occurred in the MPS in vitro and in vivo. To mimic the physiological environment in peripheral circulation after cryo-thermal therapy, 10% serum from cryo-thermal-treated mice were added in vitro co-culture with sEVs and PBMCs. Therefore, the low uptake of labelled EVs as shown in Figure 2B would be due to the addition of sEVs (obtained from serum and afterwards labelled) competing with the ones present on the 10% serum media.

Supplementation with sEVs released after cryo-thermal therapy resulted in a pronounced increase in the population of macrophages. Macrophages are innate immune cells that undergo M1 polarization earlier after cryo-thermal therapy than DCs [27]. Moreover, MDSCs can acquire a macrophage phenotype and perform antitumor immune functions in an inflammatory environment [42,43,44]. In this study, the level of MDSCs was obviously decreased, and the expression of the macrophage marker F4/80 was significantly upregulated in MDSCs and monocytes, suggesting the differentiation of monocytes into macrophages. After cryo-thermal therapy, supplementation with sEVs bearing tumor antigens and danger signals may have provided extra immunostimulatory signals to pattern-recognition receptors on these myeloid cells to activate innate immunity [43,44].

Moreover, sEV supplementation after cryo-thermal therapy promoted the differentiation of CD4^+^ T cells into the Th1 subset at an early stage after cryo-thermal therapy. The early established Th1 population can be largely maintained despite new inflammatory cues [45]. CD4^+^ Th1 cells have been found to be highly cytotoxic and equally capable of directly killing tumor cells as CD8^+^ T cells [46]. These cells exert killing effects by directly recognizing MHC II-expressing tumor cells in a B cell- and NK cell-independent manner or through interactions with tumor-infiltrating macrophages [47,48,49]. In addition, Th1 cells can recruit innate and adaptive immune cells and coordinate their differentiation [50]. A cognate interaction between tumor antigen-specific CD4^+^ Th1 cells and macrophages can shift the intratumoral M1/M2 ratio toward M1 [51]. Moreover, Th1 cells can prime antigen-specific CD8^+^ T cells, and Th1-related cytokine IFN-γ production can inhibit the development of Tregs [52,53]. Previously, we reported that the lasting antitumor immunity induced by cryo-thermal therapy is mediated by CD4^+^ Th1 cells [19,54]. Thus, CD4^+^ Th1 cells could facilitate the lasting antitumor immune response that was enhanced by the combination of cryo-thermal therapy and supplementation with sEVs.

However, the issue of why supplementation with sEVs after cryo-thermal therapy could trigger the Th1 differentiation of CD4^+^ T cells should be addressed. sEVs released after cryo-thermal therapy provided additional tumor antigens. A high-antigen affinity positively correlates with Th1 differentiation [55]. Moreover, the decreased proportion of MDSCs after supplementation with sEVs after treatment would be one of the underlying mechanisms. MDSCs can inhibit the differentiation of tumor-specific CD4^+^ T cells into CD4^+^ Th1 cells through IL-6 production [56]. Studies have also revealed that declines in MDSCs correlate with a shift in the Th1/Th2 balance toward the Th1 response [57]. Furthermore, heat-stressed EVs transporting danger signals can activate myeloid cells and promote CD4^+^ T cell secretion of the Th1-related cytokine IFN-γ [21,22]. Finally, monocyte transformation into macrophages after sEV supplementation further supports the differentiation of CD4^+^ T cells into the CD4^+^ Th1 subpopulation. M1-dominant macrophages can stimulate naïve T cells to induce Th1 cytotoxic responses [58]. Our previous work reported that M1 macrophage polarization after cryo-thermal therapy induces the differentiation of CD4^+^ T cells into Th1 and CD4 CTLs, which mediate long-term antitumor immune responses [27]. Whether the differentiation of CD4^+^ T cells to Th1 cells after sEV supplementation was due to increased numbers of macrophages deserves further examination.

In this study, we found a significant upregulation in the proportion of Th17 cells after cryo-thermal therapy, but the proportion of Th1 cells was not significantly changed. In contrast, supplementation with sEVs decreased the proportion of Th17 cells but increased the proportion of Th1 cells, which was critically important for inducing antitumor cellular immunity. These results suggested that the combination of sEVs and cryo-thermal therapy might drive the Th1/Th17 balance to the Th1 subset. It is reported that IL-23 and IL-12 can drive the transition from Th17 to Th1 cells [59]. However, whether these cytokines regulated the Th1/Th17 balance after cryo-thermal therapy combined with sEV supplementation should be further studied.

According to our previous studies, changes in immune cells were triggered on day 3 after cryo-thermal therapy (unpublished data). To investigate whether supplementation with sEVs could promote antitumor immunity induced by cryo-thermal therapy, immune cells in the spleen and peripheral blood were analyzed on day 3. This study aimed to examine how enriched sEVs could improve the efficacy of cryo-thermal therapy, so the detailed sources and packaged cargos of sEVs should be studied in the near future.

The co-isolation of serum lipoproteins could not be excluded by ultra-centrifugation [60], which could be in part responsible for the anti-tumoral effects. A combination of several methods is reported to remove different co-isolated lipoproteins [61,62] when the sample volume is large enough [60]. However, these methods were not feasible in this study because of the small volume of mice serum.

In summary, cryo-thermal therapy induced the release of large amounts of sEVs carrying antigens and danger signals into circulation. sEVs interacted with immune cells and were mainly phagocytosed by monocytes and macrophage-like monocytes in the periphery. In highly malignant 4T1 breast tumors, posttreatment supplementation with sEVs released after therapy could effectively orchestrate the differentiation of MDSCs and monocytes into macrophages and CD4^+^ T cells toward the Th1 subtype; thus, enhancing the efficacy of cryo-thermal therapy. EVs are remarkably biocompatible, and several EV-based therapies have already made their way from the bench to the bedside [63]. Isolated sEVs from cryo-thermal therapy-treated patients may be clinically applied in combination with cryo-thermal therapy to enhance the outcome of patients with malignancies.

## 4. Materials and Methods

### 4.1. Cell Culture

The murine mammary carcinoma 4T1 cell line was provided by Shanghai First People’s Hospital, China. The mouse melanoma tumor B16F10 cell line and the murine derived macrophage RAW264.7 cell line were kindly provided by Prof. Weihai Yin and Xiaoni Kong (Shanghai Jiao Tong University, Shanghai, China). Cells were cultured in Dulbecco’s Modified Eagle Medium (GE Healthcare, Logan, UT, USA) with 10% fetal bovine serum (ScienCell, Carlsbad, CA, USA), 100 U/mL penicillin, and 100 µg/mL streptomycin (HyClone, Logan, UT, USA) at 37 °C in a humidified 5% CO_2_ incubator.

### 4.2. Animal Models

All animal experiments were approved by the Animal Welfare Committee of Shanghai Jiao Tong University, and the experimental methods were performed in accordance with the guidelines of Shanghai Jiao Tong University Animal Care (approved by Shanghai Jiao Tong University Scientific ethics committee). Female BALB/c and C57BL/6 mice were obtained from Shanghai SLAC Laboratory Animal Co., Ltd., Shanghai, China, then housed and fed sterile food and water with standard nutritional formula in isolated cages of 12 h light/dark cycle. The 4T1 cells (4 × 10^5^) or B16F10 cells (5 × 10^5^) were inoculated subcutaneously at the right femoral region of the mice (at the age of 6–8 weeks) and monitored every day. Eighteen days (4T1 model) or twelve days (B16F10 model) after tumor inoculation, when the average tumor size reached 0.25 cm^3^, the mice were treated with cryo-thermal therapy. The volume of tumors was calculated using the Formula: *V* (cm^3^) = *π*/6 × *L* (*major axis*) × *W* (*minor axis*) × *H* (*vertical axis*).

### 4.3. Cryo-Thermal Therapy Procedure

The cryo-thermal therapy procedure was carried out as previously reported [14,15,16]. Briefly, the cryo-thermal therapy system was composed of liquid nitrogen for cooling and radiofrequency for heating. A compatible probe was designed with a concave-shaped tip of 10 mm in diameter to suit the size of mouse tumors, reducing contact thermal resistance. The primary rapid freezing process was at the temperature of −20 °C for 5 min, followed by radiofrequency heating at the temperature of 50 °C for 10 min after rewarming. Before the treatment, mice were anesthetized with 5% trichloroacetaldehyde hydrate (1 mL/100 g, i.p.) and tumor sites were sanitized with alcohol and iodine tincture. All the procedures were carried out aseptically.

### 4.4. Isolation of Splenic Macrophages, DCs and MDSCs

The spleens were harvested after cryo-thermal therapy. Splenocytes were separated by Gentle MACS dissociator (Miltenyi Biotec, Bergisch Gladbach, Germany), following the removal of RBCs and tissue debris. CD68^+^ (PE) macrophages and Gr1^+^ (PE) MDSCs were isolated through PE selection kit (STEMCELL, Vancouver, BC, Canada). DCs were isolated through CD11c^+^ selection kit (STEMCELL). The isolated cells with a purity of >90% were used for experiments.

### 4.5. RNA Extraction and Real-Time PCR

Total RNA was extracted from cells using TRIzol Reagent (Takara Bio, Mountain View, CA, USA). cDNA was obtained using PrimerScript RT reagent kit (Takara Bio, Mountain View, CA, USA). The SYBR Premix Ex Taq (Takara Bio, Mountain View, CA, USA) and cDNA samples were amplified in 384-well plates. Quantitative real-time PCR (qRT-PCR) was performed on ABI 7900HT sequence detection system and SDS software (Applied Biosystems, Foster City, CA, USA). Relative expression levels of mRNA were normalized to glyceraldehyde 3-phosphate determined by the Ct value and assessed using relative quantification (ΔΔCt method). The following forward (F) and reverse (R) primers were used (Table 1).

### 4.6. sEVs Isolation and Characterization

sEVs were isolated and characterized accordingly [30,64]. After cryo-thermal therapy, mice serum was collected and diluted with an equal volume of PBS. Diluted serum was sequentially centrifuged at 2000 *g* for 30 min and 10,000 *g* for 45 min to remove cell debris. The supernatant was further diluted in PBS at 1:8 and pelleted by ultracentrifugation at 110,000 *g* for 2 h (Beckman Coulter OptimaL-100 XP, Brea, CA, USA). The precipitate was resuspended in a large volume of PBS and recovered by centrifugation at 110,000 *g* for 70 min. The pellets were resuspended in PBS and stored at −80 °C. The total protein concentration of sEVs was quantified by Bicinchoninic acid (BCA) assay (Thermo Fisher Scientific, Waltham, MA, USA). The particle sizes of sEVs were determined by dynamic light scattering using Malvern Zetasizer Nano (Malvern, Malvern, UK). The morphology of sEVs were observed with transmission electron microscopy (Talos L120C G2, FEI Company, Hillsboro, OR, USA). The particle size was measured by Nanoparticle Tracking Analysis (NTA, ZetaView, Particle Metrix, Mebane, NC, USA) using machine software (ZetaView 8.03.04.01). Control and 3 h sEVs were diluted 300 times and 1000 times, respectively, to reach the measurable range. Data were obtained from 5 replicates.

For Western blot analysis, 5 or 15 μg of total sEVs quantified with BCA were separated onto 10% gradient Tris-Glycine precast gels (Epizyme, Shanghai, China) and transferred to PVDF membranes (Merck Millipore, Darmstadt, Germany). Blot was probed with anti-CD63, anti-ALIX, anti-HMGB1, anti-TRP2 (Abcam, Cambridge, UK), anti-CD61 (Beyotime, Jiangsu, China) and anti-HSP70 (Cell Signaling Technology, Danvers, MA, USA). Each result was a representative from three separate experiments.

### 4.7. Phagocytosis Assay

PBMCs from tumor-bearing mice were cultured in Dulbecco’s Modified Eagle Medium (GE Healthcare, Logan, UT, USA) with 10% mouse serum from cryo-thermal-treated mice, 100 U/mL penicillin, and 100 µg/mL streptomycin (HyClone, Logan, UT, USA) at 37 °C in a humidified 5% CO_2_ incubator. sEVs were labeled with DiO (Beyotime) at a concentration of 10 μM and cocultured with PBMCs at 20 μg/mL (3.87 × 10^8^ particles/mL) from tumor-bearing mice. A total of 24 h later, the uptake of sEVs was determined by flow cytometry. For in vivo studies, 50 μg (9.68 × 10^8^ particles) of sEVs was labeled with DiI at a concentration of 10 μM (Beyotime) and given to each mouse immediately after cryo-thermal therapy procedures through the tail vein. Mice were sacrificed after 3 h. PBMCs were isolated and fixed with DAPI (Dojindo, Kumamoto, Japan) before observation with laser confocal microscope (Leica TCS SP5, Wetzlar, Germany). For in vitro studies, RAW 264.7 cells were stained with CFSE (Vector Laboratories, Burlingame, CA, USA) and seeded in 24-well plates with DiI-labeled sEVs at 20 μg/mL (3.87 × 10^8^ particles/mL) for 2–4 h. Cells were fixed with DAPI and observed (Leica TCS SP5). PBS was dyed, diluted, and centrifuged as negative control.

### 4.8. Flow Cytometry Analysis

Mice were sacrificed on day 3 after the treatments (*n* = 4 per group). Spleens were dissociated and filtered through 70 μm filters (Falcon, Corning, NY, USA) to prepare single-cell suspension of splenocytes. PBMCs were gently separated with Mouse 1 × Lymphocyte Separation Medium (Dakewe, Beijing, China). Red blood cells were removed using erythrocyte-lysing reagent containing 0.15 M NH_4_Cl, 1.0 M KHCO_3_, and 0.1 mM Na_2_EDTA. For intracellular and intranuclear staining, cells were further fixed and permeabilized after surface staining. Cells were stained with fluorescent labeled antibodies for 20 min or 45 min for transcription factors at room temperature. The staining antibodies were purchased from BioLegend (San Diego, CA, USA), including: Zombie Aqua Fixable Viability Kit, CD11b-Pacific Blue (clone M1/70), CD11c-Alexa Fluor 488 (clone N418), F4/80-APC (clone BM8), Gr-1-PE (clone RB6-8C5), Ly6G-PE/Cy7 (clone 1A8), Ly6C-FITC (clone HK1.4), CD86-APC/Cy7 (clone GL-1), I-A/I-E-PerCP/Cy5.5 (clone M5/114.15.2), CD3-PerCP/Cy5.5 (clone 145–2C11), CD4-APC/Cy7 (clone RM4–5), CD8-Pacific Blue (clone 53–6.7), T-bet-PE/Dazzle 594 (clone 4B10), FOXP3-PE (clone MF-14), Bcl6-Brilliant Violet 421 (clone K112–91), Thpok-Alexa Fluor 647 (clone T43–94), IFN-γ-PE/Dazzle 594 (clone XMG1.2), Granzyme B-Alexa Fluor 647 (clone GB11), Perforin-PE (clone S16009A), IL-4-Brilliant Violet 421 (clone 11B11), and IL-17-PE (clone TC11–18H10.1). Data were acquired on FACS Aria II flow cytometer (BD, Franklin Lakes, NJ, USA) and analyzed using FlowJo V.10 software (FlowJo LLC, Ashland, OR, USA). Negative and fluorescence minus one (FMO) control were used to indicate background staining. Immune cell populations were identified as follows (gated on live cells): Macrophages: CD11b^+^ F4/80^+^; DCs: CD11c^+^; MDSCs: CD11b^+^ Gr1^+^; Monocytes: CD11b^+^ Ly6G^−^ Ly6C^+^; CD4^+^ T cells: CD3^+^ CD4^+^; CD8^+^ T cells: CD3^+^ CD8^+^. Detailed gating strategies can be found in the Supplementary Materials (Supplementary Figures S5 and S6).

### 4.9. Statistical Analysis

The statistics were analyzed using one-way ANOVA and Student’s *t*-test on Graph Pad Prism 8 (La Jolla, CA, USA). Results were shown as mean ± SD. * *p* < 0.05, ** *p* < 0.01, *** *p* < 0.001, and **** *p* < 0.0001. Significant differences in survival were determined using a log-rank (Mantel–Cox) test.

## Figures and Tables

**Table 1:** Forward (F) and Reverse (R) primers used in Real-Time PCR.

Name	Primer Sequence (5′–3′)
GAPDH F	AGGTCGGTGTGAACGGATTTG
GAPDH R	GGGGTCGTTGATGGCAACA
ITGAV F	CCGTGGACTTCTTCGAGCC
ITGAV R	CTGTTGAATCAAACTCAATGGGC
ITGB3 F	CCACACGAAACTC
ITGB3 R	CTTCAGGTTACATCGGGGTGA
ITGAL F	CCAGACTTTTGCTACTGGGAC
ITGAL R	GCTTGTTCGGCAGTGATAGAG
ICAM1 F	TGCCTCTGAAGCTCGGATATAC
ICAM1 R	TCTGTCGAACTCCTCAGTCAC
MFGE8 F	CCGCGTCTGGTGACTTCTG
MFGE8 R	TCCTCTCTCAGTCTCATTGCAC
CXCL10 F	CCAAGTGCTGCCGTCATTTTC
CXCL10 R	GGCTCGCAGGGATGATTTCAA
CD86 F	GAGCTGGTAGTATTTTGGCAGG
CD86 R	GGCCCAGGTACTTGGCATT
MHC II F	AGCCCCATCACTGTGGAGT
MHC II R	GATGCCGCTCAACATCTTGC
IL-1β F	ACAGCAGCACATCAACAAGAG
IL-1β R	ATGGGAACGTCACACACCAG
IL-12p40 F	TGGTTTGCCATCGTTTTGCTG
IL-12p40 R	ACAGGTGAGGTTCACTGTTTCT
IL-15 F	AGAGGCCAACTGGATAGATGT
IL-15 R	AGAGCACGTTTCTTACTGTTTCA
TNFα F	TTCTGTCTACTGAACTTCGGGGTGATCGGTCC
TNFα R	GTATGAGATAGCAAATCGGCTGACGGTGTGGG
VEGFR2 F	TTTGGCAAATACAACCCTTCAGA
VEGFR2 R	GCAGAAGATACTGTCACCACC
IL-6 F	GACAAAGCCAGAGTCCTTCAGAGAGATACAG
IL-6 R	TTGGATGGTCTTGGTCCTTAGCCAC
IL-10 F	GCTCTTACTGACTGGCATGAG
IL-10 R	CGCAGCTCTAGGAGCATGTG
IL-4R F	CCCCAGCTAGTTGTCATCCTG
IL-4R R	CAAGTGATTTTTGTCGCATCCG
TGF-β F	CTCCCGTGGCTTCTAGTGC
TGF-β R	GCCTTAGTTTGGACAGGATCTG

## Data Availability

Data are contained within the article or Supplementary Materials or are available from the authors upon reasonable request.

## Supplementary Materials

The following are available online at https://www.mdpi.com/article/10.3390/ijms222011021/s1.
