# Supplementary material for: Supplementation with Serum-Derived Extracellular Vesicles Reinforces Antitumor Immunity Induced by Cryo-Thermal Therapy
Source: Int J Mol Sci. 2021 Oct 13;22(20):11021. doi: 10.3390/ijms222011021 (PMC8539038; doi:10.3390/ijms222011021)
Supplement: Supplementary file 1 [file ijms-22-11021-s001.zip › ijms-1371517-supplementary.pdf]

International Journal of *Molecular Sciences*

MDPI

Supplementary Materials: Supplementation with serum-derived extracellular vesicles reinforces antitumor immunity induced by cryo-thermal therapy

Yinuo Cen [1,2], Yue Lou [1,2], Junjun Wang [1,2], Shicheng Wang [1,2], Peng Peng [1,2], Aili Zhang [1,2] and Ping Liu [1,2,*]

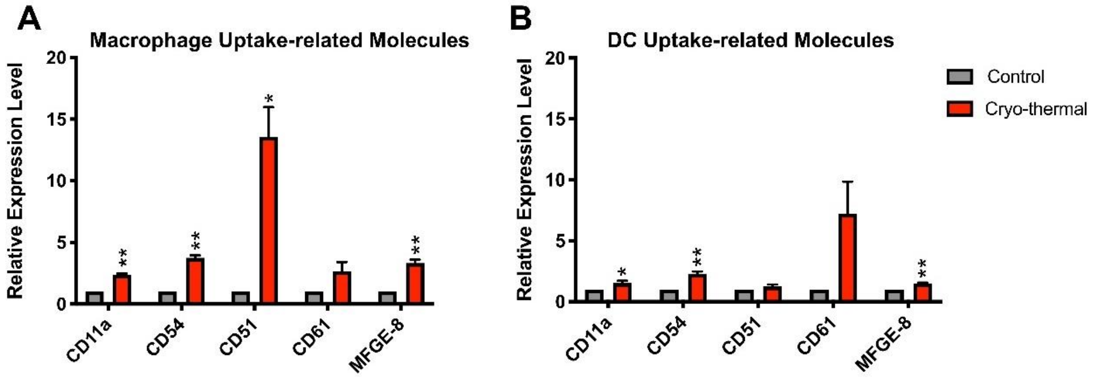

Supplementary Figure S1 The up-regulated expression of EV uptake-related genes in spleen macrophages and DCs on day 3 after cryo-thermal therapy in 4T1 subcutaneous tumor model. Total RNA of spleen macrophages and DCs were isolated on day 3 after treatment. RT-qPCR was used to detect mRNA levels of EV phagocytosis-related protein of macrophages (**A**) and DCs (**B**) on day 3 after treatment. Data were shown as mean±SD, and the student's t-test was used for statistical analysis. n = 4, * $p < 0.05$, ** $p < 0.01$ compared with the control group.

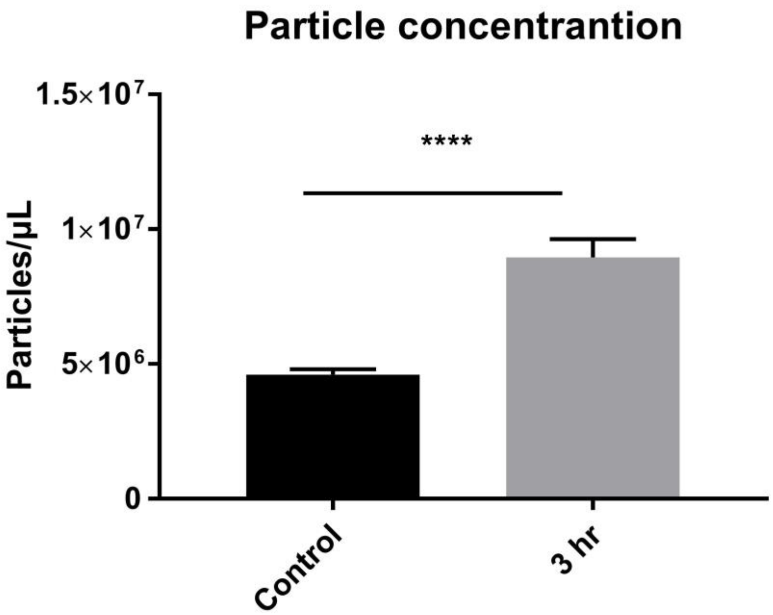

Supplementary Figure S2 Cryo-thermal therapy induced the release of sEVs. sEVs concentration (standardized by serum volume) 3hr after cryo-thermal therapy were measured by Nanoparticle Tracking Analysis (NTA). Data were shown as mean±SD, and the student's t-test was used for statistical analysis. n = 5, **** $p < 0.0001$ compared with the control group.

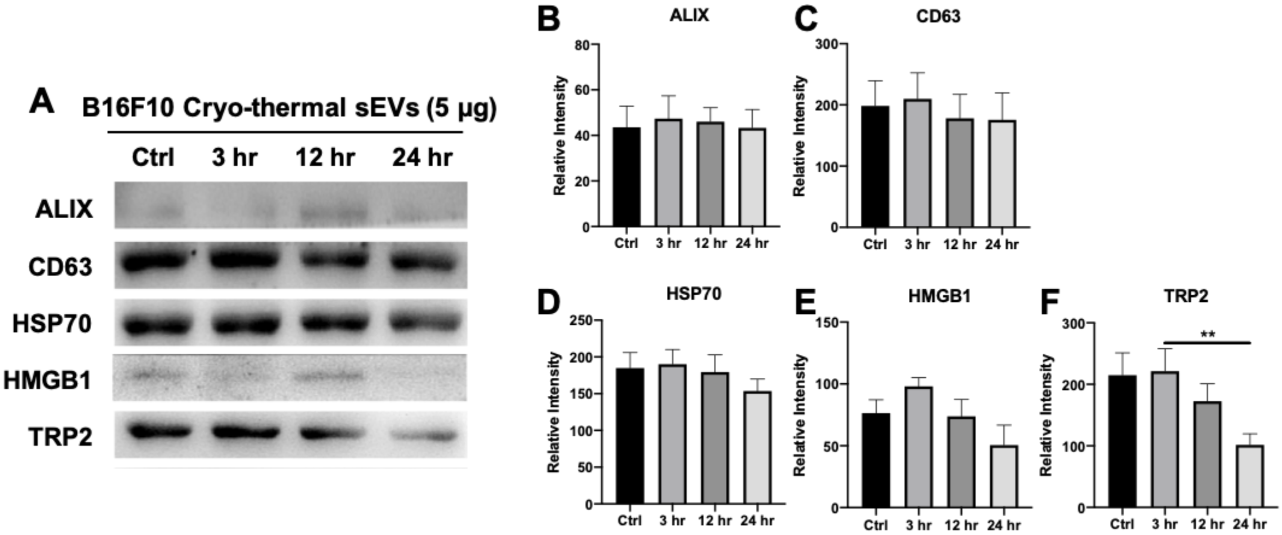

Supplementary Figure S3 Western blotting of sEVs (5 μg) from the serum in B16F10 model at 3, 12, and 24 hr after cryo-thermal therapy with individual densitometry analysis. ALIX, CD63: Exosomal protein markers; HSP70, HMGB1: danger signal; TRP2: known B16 tumor antigen. Data were shown as mean±SD, and the student's t-test was used for statistical analysis. n = 3, ** $p < 0.01$.

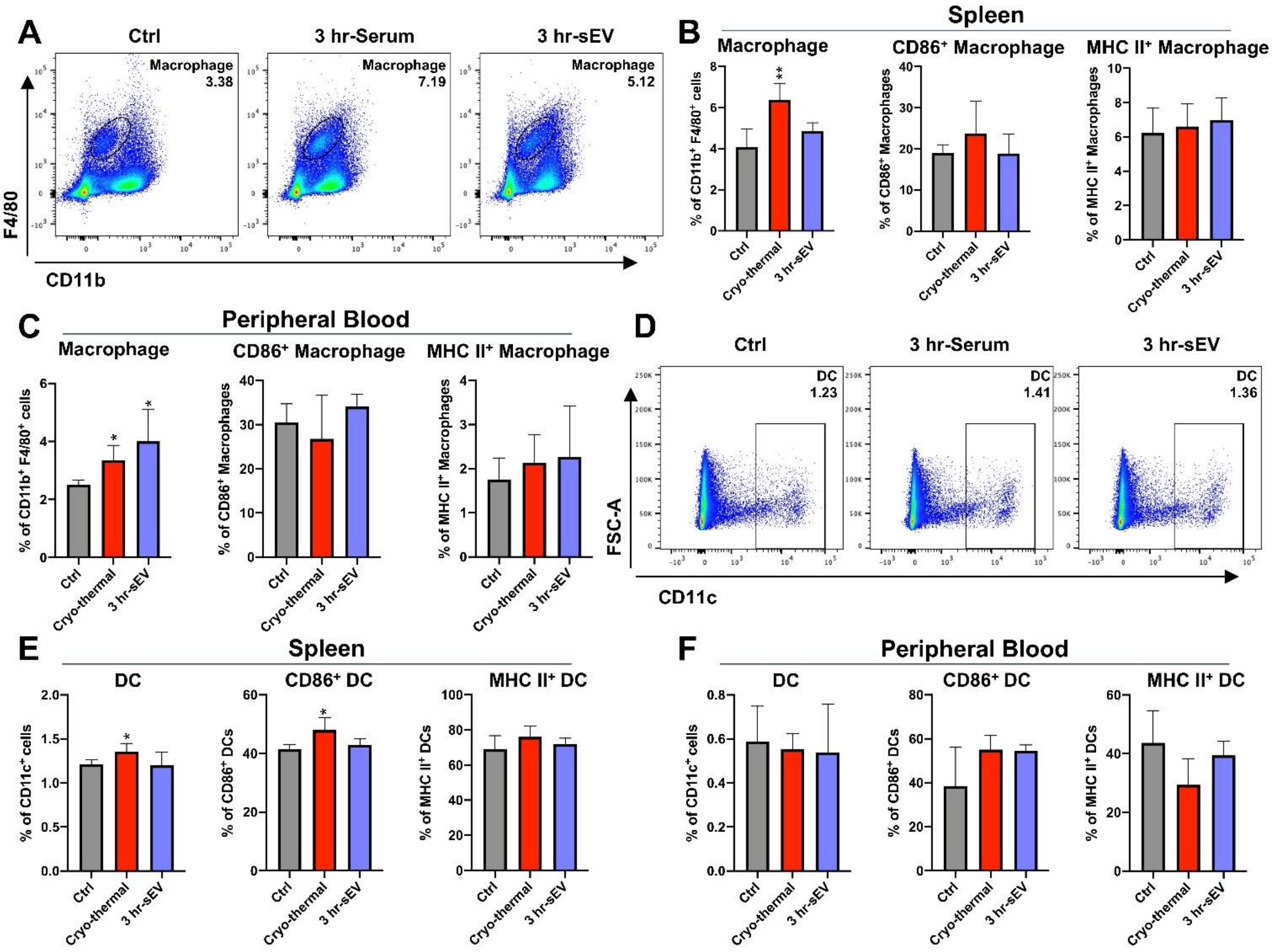

Supplementary Figure S4 Supplement of sEVs after cryo-thermal therapy upregulated the proportion of macrophages and DCs. (**A**) Flow cytometry gating strategy for the determination of CD11b⁺ F4/80⁺ macrophages. (**B**-**C**) Proportions of macrophages, CD86⁺ macrophages, and MHC II⁺ macrophages in spleen (**B**) and peripheral blood (**C**)were determined by using flow cytometry (n = 4 per group). (**D**) Flow cytometry gating strategy for the determination of CD11c⁺ DCs. (**E**-**F**) Proportions of DCs, CD86⁺ DCs, and MHC II ⁺ DCs in spleen (**E**) and peripheral blood (**F**) were determined by using flow cytometry (n = 4 per group). Data were shown as mean±SD, and the student's t-test was used for statistical analysis. * $p < 0.05$, ** $p < 0.01$ compared with the control group.

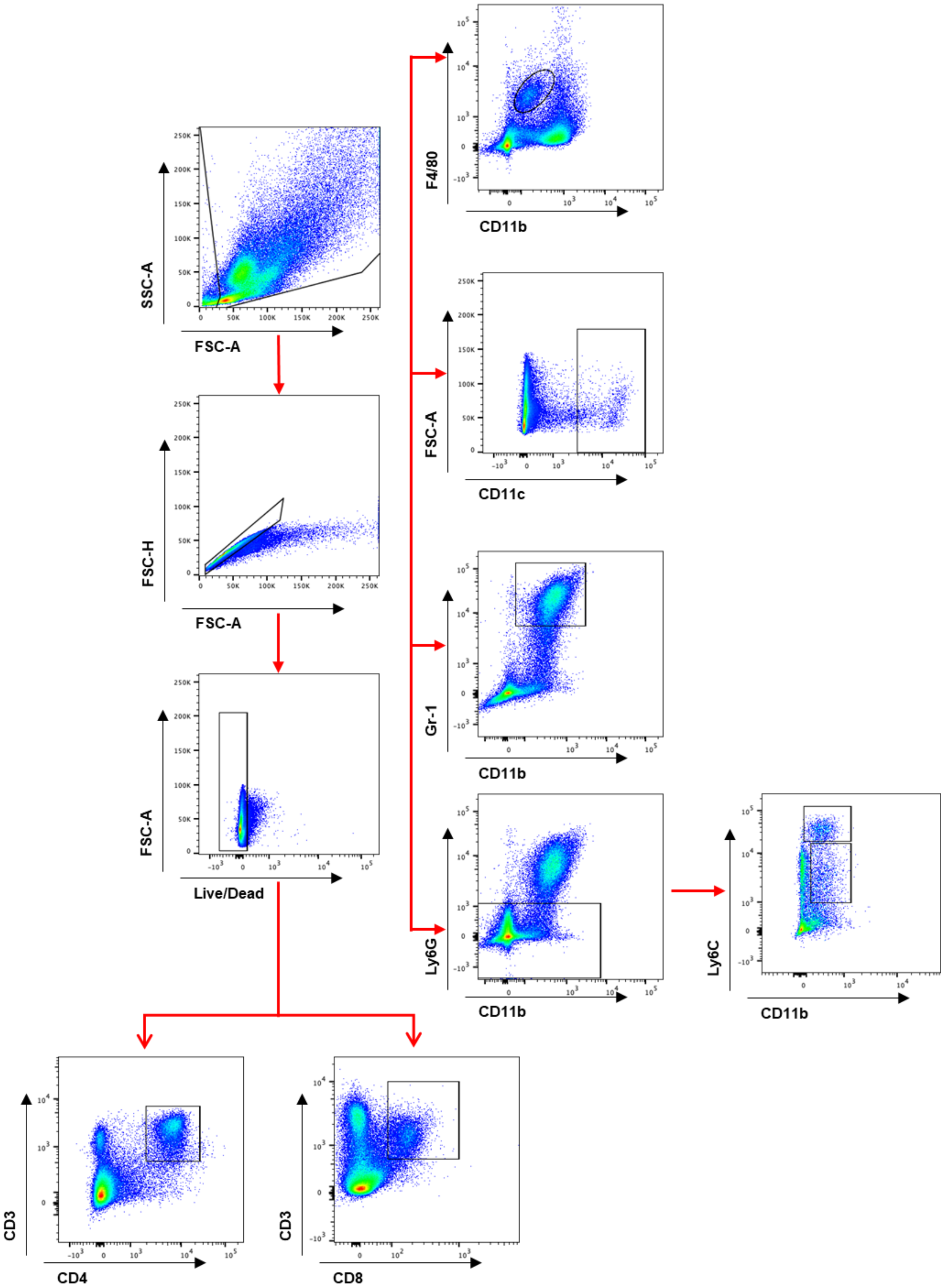

Supplementary Figure S5 Flow cytometry gating strategy for the determination of CD11b⁺ F4/80⁺ macrophages, CD11c⁺ DCs, CD11b⁺ Gr1⁺ MDSCs, CD11b⁺ Ly6G⁻ Ly6C⁺ Monocytes, CD3⁺ CD4⁺ T cells and CD3⁺ CD8⁺ T cells in in vivo analyses.

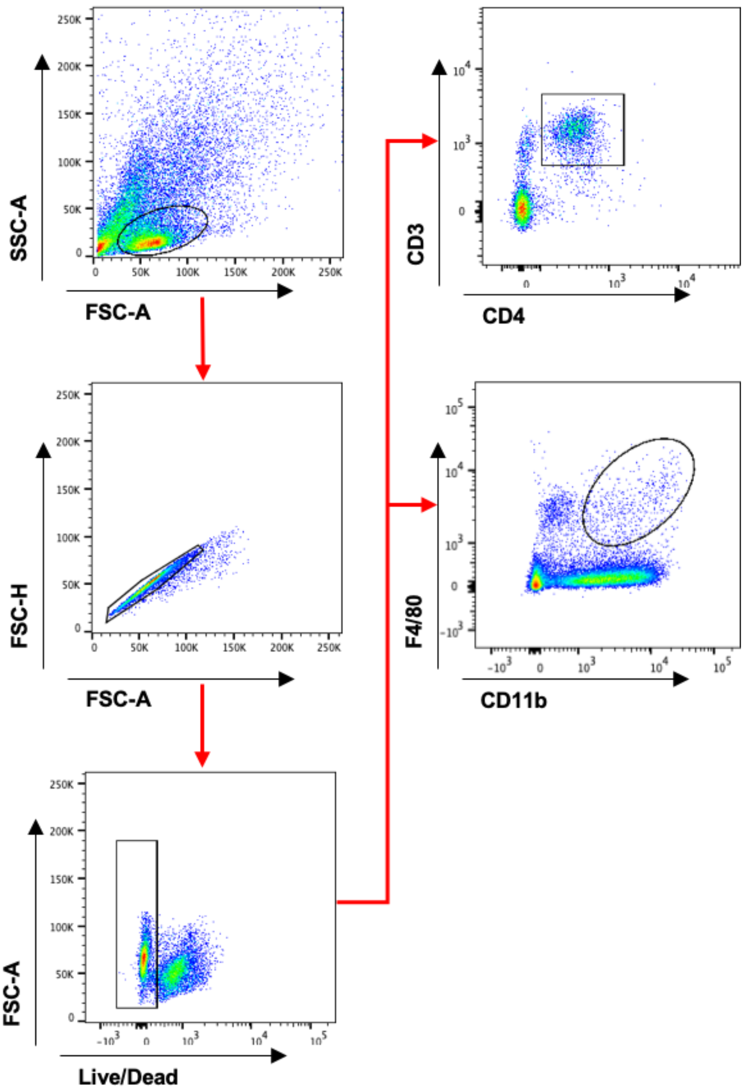

Supplementary Figure S6 Flow cytometry gating strategy for the determination of CD11b+ F4/80+ macrophages and CD3+ CD4+ T cells in in vitro analyses.
